# How to Strengthen Wildlife Surveillance to Support Freedom From Disease: Example of ASF Surveillance in France, at the Border With an Infected Area

**DOI:** 10.3389/fvets.2021.647439

**Published:** 2021-06-08

**Authors:** Stéphanie Desvaux, Christophe Urbaniak, Thibaut Petit, Pauline Chaigneau, Guillaume Gerbier, Anouk Decors, Edouard Reveillaud, Jean-Yves Chollet, Geoffrey Petit, Eva Faure, Sophie Rossi

**Affiliations:** ^1^French Agency for Biodiversity (OFB), Wildlife Health Unit, Birieux, France; ^2^Regional Hunters' Federation (FRC), Châlons-en-Champagne, France; ^3^French Agency for Biodiversity (OFB), Regional Delegation, Rozerieulles, France; ^4^National Hunters' Federation (FNC), Issy-les-Moulineaux, France; ^5^French General Directorate for Food (DGAL), Animal Health Unit, Strasbourg, France; ^6^French Agency for Biodiversity (OFB), Wildlife Health Unit, Orléans, France; ^7^French General Directorate for Food (DGAL), Animal Health Unit, Bordeaux, France; ^8^French Agency for Biodiversity (OFB), Wildlife Health Unit, Auffargis, France; ^9^French Agency for Biodiversity (OFB), Wildlife Health Unit, Gap, France

**Keywords:** surveillance, wild species, African swine fever, freedom from animal disease, cadaver detection dogs

## Abstract

Using a risk-based approach, the SAGIR network (dedicated to wildlife disease surveillance) had to strengthen surveillance activities after ASF was confirmed in Belgium in September 2018, very near the French border. Three new active dead wild boars search protocols supplemented opportunistic surveillance in Level III risk areas: patrols by volunteer hunters, professional systematic combing, and dog detection. Those protocols were targeted in terms of location and time and complemented each other. The main objectives of the designed surveillance system were (i) to assure early detection in case of introduction of the disease and (ii) to support the free status of the zone. Compiling the surveillance effort was thus a necessity to assure authorities and producer representatives that the sometimes low number of carcasses detected was not a consequence of no surveillance activities. The human involvement in implementing those activities was significant: more than 1000 8-h days just for the time spent in the field on active search activities. We calculated a specific indicator to enable a comparison of the surveillance results from different zones, including non-infected Belgian zones with strengthened surveillance activities. This was a first step in the evaluation of the efficacy of our surveillance activities in a WB population. Field experiments and modelling dead WB detection probability are planned to supplement this evaluation. Belgium regained its ASF-free status in November 2020, and ASF was not detected in France in either the WB or domestic pig populations.

## Introduction

Since 1986, a network dedicated to wildlife disease surveillance called SAGIR has been in place in France (mainland and overseas territories). SAGIR is a participatory network organising an event-based surveillance, which aims at detecting the principal causes of wildlife mortality (1). The French Hunting and Wildlife Agency (ONCFS) is responsible for the scientific coordination of the SAGIR network [the ONCFS became the French Agency for Biodiversity (OFB) in January 2020].

As African Swine Fever (ASF) spread in Eastern Europe between 2014 and 2018, the level of vigilance was progressively raised within the SAGIR network in France, but no specific area of the territory was assumed to be at higher risk of introduction. The detection of ASF in the wild boar (WB) population in Belgium around 10 km from the French border in September 2018 (2) directly impacted SAGIR's activities. During the first weeks, it was not known how long the disease had been circulating and whether the disease was only concentrated in the Etalle forest where it had been initially detected. French authorities immediately decided to ban hunting in 134 municipalities at the border in order to avoid WB movement and take the time to get a better understanding of the disease distribution (hunting was progressively re-opened from October 20, 2018). Access to forests was also restricted. In this context, the presence of the usual observers of wildlife mortality (e.g., hunters and foresters) was limited, undermining the chance to receive reporting on observed dead WB.

From September 2018, the SAGIR network's objectives in the area bordering Belgium were (i) to assure early detection in case of introduction of ASF and (ii) to support the free status of the zone. To early detect the disease, SAGIR had to detect, sample, and test as many WB carcasses as possible (roadkill included). As the movement restrictions and the hunting ban reduce the chances for passive surveillance, surveillance reinforcement through active carcass search was proposed. Protocols that assured professional and voluntary observation in good biosecurity conditions were developed for three types of searches: (i) hunter patrols, (ii) systematic combing of forest in at-risk forests, and (iii) dog detection. From mid-February 2019, an active surveillance program was also conducted. Twenty percent of the hunted WBs were sampled and tested by RT-PCR (data not presented). We also developed a procedure for dating carcasses (with the support of forensic police) in case of confirmed infection.

Documenting freedom from disease in a wild population is a methodological challenge. Although hunting bags may be used as a proxy for the WB population, it is impossible to know how many naturally dead WBs are present in a territory during a specific period and, as a consequence, how many of them the surveillance activities should detect. In a crisis context, “no carcass” may be understood as “no surveillance” by authorities or producer representatives. It quickly became necessary to collect and document the surveillance effort, in particular for active searches. Surveillance purely event-based was impossible to measure, as it is based on a high number of field actors performing activities not specifically dedicated to surveillance.

In this article, we describe how ASF surveillance activities were conducted from September 2018 to the end of August 2020 in the wild boar population of the region at the border with Belgium. We analysed the surveillance effort for each of the surveillance modalities in order to learn lessons in terms of human resource management in a context of high risk of introduction in an area. We have also developed an indicator to compare surveillance efficacy between zones (in France and in Belgium). Finally, we discussed how those activities contributed to document freedom of disease in the WB population at the border with Belgium.

## Materials and Methods

### Study Area

**Figure 1:**
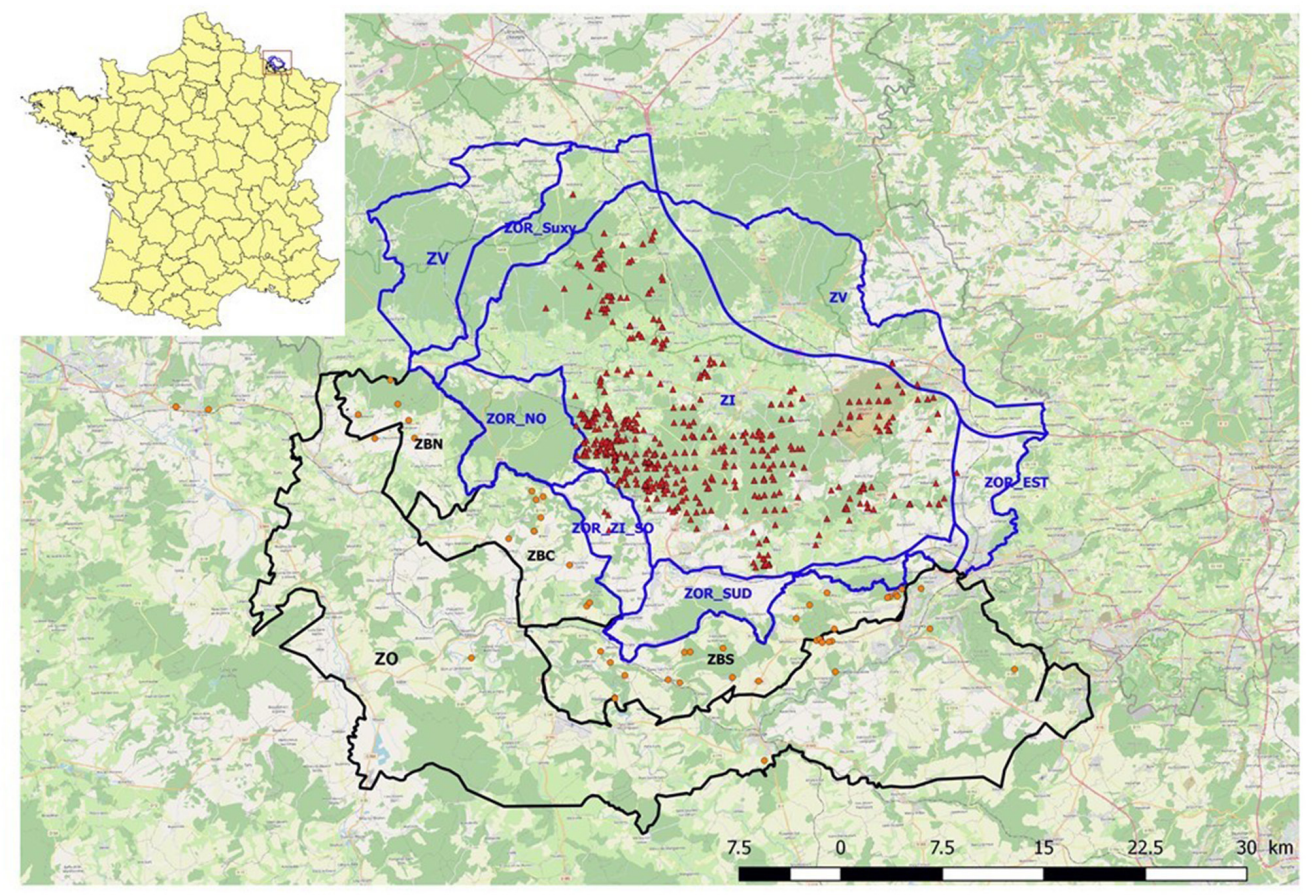
Regulated zones in France (black line, with ZO = Level II, ZBN, ZNC, and ZBS = Level III) and in Belgium (blue line) as defined in April 2019 with all the carcasses collected in the French Level III areas (orange dots) and in the infected Belgian area (red triangle—source OIE) during the study period (September 2018 to August 2020).

In September 2018, the French metropolitan territory was divided into three areas using a risk-based surveillance approach (3):

- Level III: infected area or area where infection is suspected (ZBN, ZBC, and ZBS in Figure 1),
- Level II B: increased risk of introduction due to proximity (i.e., neighbouring an infected area or an area where infection is suspected) (ZO in Figure 1, as well as Corsica Island for its proximity to Sardinia),
- Level II A: increased risk of introduction by long- or medium-distance transmission (the rest of the territory).

No part of metropolitan France was kept in Level I, as this level is the base level in a context of low risk of introduction. Surveillance efforts were thus distributed differently in these three areas. Active search activities were only implemented in the Level III area, including 134 (from 15/09/2018 to 19/10/2018), then 50 municipalities at the border with Belgium (Figure 1 shows the regulated zones in France and Belgium as defined in April 2019). In January 2019, a depopulation zone (with intense WB destruction activities) was defined within the Level III area. Fences were built progressively to separate this depopulation zone, which in the end overlapped the Level III area. The surface area of the Level III area, as defined in Figure 1, was about 300 km^2^, divided into three fenced zones (ZBN, ZBC, and ZBS).

### Description of the Three Active Carcass Search Protocols

The three protocols for active carcass searches were designed to complement each other in terms of location and time and to supplement opportunist surveillance. Contrary to opportunistic surveillance, they allow to target areas at higher risk of introduction.

Hunter patrols were organised rapidly in September 2018. These were initially planned for a few weeks to help assess the epidemiological situation at the border with Belgium. This activity targeted municipalities at the border with the infected Belgian area (*n* = 27). The objective was to have at least one hunter patrol per week in each hunting ground. Hunters had to organise a search (prospecting), targeting areas with known WB presence based on their experience of the past few years and their field observations. The route should include, if present:

- mud and water holes (known to be commonly used by WBs) and feeding grounds as areas known to be attractive to WBs;
- fences and valley where WBs may look for small rivers. Indeed, we know that infected WBs will look for water (because of the fever) and may be more easily stopped by physical barriers and be unable to escape. As a consequence, they may be found dead along fences (4–6).

We rapidly decided to offer the volunteer hunters financial compensation (30 euros per field session).

Systematic combing of forests, unlike hunter patrols, aimed to cover the entirety of a forest area using a method also implemented in Belgium: silent drive “hunt” by teams of around 10 persons. ONF (National Forest Agency) foresters were responsible for supervising the teams made up of military volunteers and ONCFS staff. We selected areas to be combed from forests in buffer zones of 5–7 km from the nearest Belgian ASF cases. Forests shared between France and Belgium were prioritised. This surveillance activity started in January 2019 and stopped in July 2019, when no new cases had been reported <7 km from the border. Between January and July 2019, they were planned every week or every 2 weeks based on a rapid risk assessment analysing the locations of the Belgian ASF cases, the forest continuity between the cases and the French border, and the presence of fences. Planning was also determined by the availability of human resources.

Dog detection was planned for the same at-risk area as the systematic combing but in different locations: at the border of the forests, close to rivers, in pastures and in areas that are difficult for humans to access. In January 2019, three dog handlers contracted by ONCFS started to train their dogs to detect the scent of dead WB, and fieldwork started 3–4 weeks later. During the study period, five other dogs were trained and used in the field. The protocol included restrictions during very hot or very cold periods. A specific biosecurity procedure was developed for this activity, including washing and disinfecting the dogs' legs after each field session (a field day was made up of several field sessions as dogs cannot search for a long time).

For each of those surveillance activities, a specific form was designed, filled out, and compiled. Hunting organisations developed a shared database and were responsible for compiling and entering data for hunter patrols. They also collected forms from foresters and entered data for systematic combing. The ONCFS compiled the dog detection and systematic combing data.

### Weekly National Reports

In addition to weekly summary reports of the number of carcasses detected in each area, we had to report the surveillance effort related to the active search activities performed in the Level III area. The spatial unit used to compile data was the hunting ground. Other authorised activities involving professionals in the field were also reported (forestry work or drive hunts during the hunting season). Although those activities were not specifically dedicated to surveillance, they were performed by people who had received information or training on ASF and knew that it was compulsory to report any dead WBs observed.

### Measuring the Surveillance Effort

To better understand the human resources needed for each activity, the surveillance effort was measured by calculating the total number of field sessions, their duration, and the total human involvement in hours (duration of each field session multiplied by the number of people involved). Surveillance effort for opportunistic surveillance was impossible to measure. We also calculated the average spatial coverage—as a line or a surface covered—for each active search activity.

### Evaluation of Surveillance Efficacy

To evaluate surveillance efficacy, we developed an indicator enabling us to roughly compare the surveillance results between zones by dividing the number of carcasses detected by a proxy for the WB population. The proxy used is the forest surface area because it is assumed to be proportional to the WB population (7, 8). The forest surface area was extracted from the CORINE Land Cover ® database (2018, vector data). Three classes of vegetation were considered: broad-leaved forest, coniferous forest, and mixed forest.

The zones to be compared included the three zones of our Level III area in France (ZBN, ZNC, and ZBS) and three zones in Belgium at the border with France (ZOR_NO, ZOR_ZI_SO, and ZOR_SUD) (Figure 1). Due to their location, the three zones in Belgium present landscape continuity with the French Level III area. Two of them (ZOR_NO and ZOR_SUD) share a similar epidemiological situation with the French zones: they were not infected but close to an infected area. They also experienced active search activities not described in this study. The zone named ZOR_ZI_SO, initially of similar status to the other two, became infected in January 2019 and regained its previous status in May 2020 (9). Data from Belgium surveillance was compiled from the reports produced and shared by the Public Service of Wallonia.

### ASF Detection

ASF RT-PCR analyses were performed by local screening laboratories. Two commercial kits were used: ADIAVET ASFV Fast Time and ID Gene ASF Duplex. In case of positivity, the sample would have been sent to the French national reference laboratory (ANSES).

## Results

### Active Search Activities and Reporting

Hunter patrols were initially planned for a few weeks to evaluate the epidemiological situation in the hunting grounds closest to the Belgian ASF cases. In the end, they were continued until the end of 2020 to support the free status of the zone as well as to guarantee early detection in case of introduction. In total, between September 2018 and August 2020, 2144 field sessions were organised with some fluctuation over the months (Table 1).

**Table 1:** Surveillance effort and carcass detection compiled by active search activity over the study period in France, at the border of an infected area.

		**Sept–Dec 18**	**Jan–Apr 19**	**May–Aug 19**	**Sept–Dec 19**	**Jan–Apr 20**	**May–Aug 20**	**TOTAL**
Number of field sessions	Hunter patrols	314	352	473	487	264	254	**2,144**
	Systematic combing	0	28	29	0	0	0	**57**
	Dog detection (in days)	0	14	26	11	10	5	**66**
	Hunter patrols	643	734	1,071	1,049	607	630	**4,734**
Total duration of the field sessions (hours)	Systematic combing	0	96	124	0	0	0	**220**
	Dog detection	0	42	59	26	26	9	**162**
	Hunter patrols	981	992	1,342	1,354	818	643	**6,130**
Total human involvement (hours)	Systematic combing	0	1,026	1,358	0	0	0	**2,384**
	Dog detection	0	100	145	59	58	21	**383**
	Hunter patrols	1	0	0	1	0	0	**2**
Number of carcasses detected	Systematic combing	0	4	0	0	0	0	**4**
	Dog detection	0	2	0	0	0	0	**2**
	SAGIR opportunistic surveillance (roadkill)	22 (17)	15 (8)	3 (2)	4	2	0	**46**

Systematic combing activities started just after an ASF case was confirmed in two WBs hunted outside the infected and fenced area in Belgium in January 2019 (located in ZOR_ZI_SO in Figure 1). Those cases increased the perceived risk of ASF introduction to France. It became even more necessary to assure no unusual mortality affected the French WB population. Rapidly, we experienced difficulties in properly exploring some landscapes, as moving forward in a line can be extremely difficult when brambles are present and during spring and summer. Furthermore, we faced some difficulty in terms of manpower. Thus, in spring 2019, we decided to target areas with higher chance of carcass detection using a model developed by the Belgian team (4). The model was applied to our Level III area, and the total surface area to comb in a forest was thus reduced by around 75%. In total, between September 2018 and August 2020, 57 systematic combing field sessions were organised.

Dog detection was initially planned to supplement systematic combing by targeting areas not easily covered by that activity. We rapidly refined our strategy in order to avoid the dogs having to search for hours in too uncomfortable environment, especially in dead nettles in springtime or in brambles. Dog detection was used from February 2019 to August 2020. In total, between September 2018 and August 2020, 66 field days (with several search sessions per day) were organised. We also had to adapt our biosecurity protocol to properly clean and disinfect the dogs' legs with appropriate products only at the end of the day and not after each search session, as this procedure was a source of stress for some of the dogs.

Reporting was organised on a weekly basis by producing tables with all the carcasses detected and tested for ASF by zone and maps compiling all the search activities at each hunting ground level. Reports were posted on the National Animal Health Surveillance Platform (NAHSP) website (www.plateforme-esa.fr).

### Estimation of the Surveillance Effort

#### Human Resources

The total human involvement for each active search activity (duration of the field sessions multiplied by the number of people involved) is given in Table 1. Its shows that hunter patrols mobilised much higher human resources than the other two activities, with a total of 6,128 h dedicated to these patrols, vs. 2,384 h and 384 h, respectively, for systematic combing and dog detection. Although hunter patrols were planned weekly, we observed some fluctuations over the months (Table 1). The other activities were planned according to the epidemiological situation.

The way the field teams were organised was also very different for the three activities. In more than 80% of cases, the hunter patrols were implemented by only one hunter (median: 1, min: 1, max: 17), whereas systematic combing was performed by a team of 11 people on average (median: 10, min: 6, max: 39). Dog detection teams were usually made up of the dog handler(s) and one person from the ONCFS (median: 2, min: 2, max: 4). The duration (in hours) of the field sessions also differed: whereas hunter patrols lasted on average 02:12 per session (median: 02:00, min: 00:15, max: 09:45) over a long period (2,144 field sessions), the field sessions for systematic combing were more limited in number (*n* = 57), but each session lasted on average 03:50 (median 04:00, min: 01:09, max: 06:50). The dog detection teams worked on 66 days from February 2019 to August 2020, with search activities lasting on average 02:30 per day (median: 02:30, min: 00:29, max: 05:40). A working day with the detection dogs was divided into small search sessions of 00:50 on average (median: 00:43) separated by resting and/or training time.

#### Spatial Coverage

Distance travelled by hunter patrols was 5.6 km on average per field session (median: 4.6 km). Systematic combing covered on average 388 ha per field session (median: 400 ha) with an average speed of 109 ha per hour (median: 104 ha). The distance travelled by dog handlers was 4.7 km on average per field day (median: 4.9 km). We calculated that the dogs covered 2.3 times more distance than the dog handlers.

### Evaluation of Surveillance Efficacy

#### Carcass Detection

In total, 54 carcasses were reported in the Level III zone, 53 were collected and tested using RT-PCR testing, and 1 was not found by the field team (detected on the roadside by an observer) (see Figure 1 for location). Among those 54 carcasses, 43 were located in the 300-km^2^ Level III area as defined in Figure 1. Eighty-seven percent of the carcasses were detected during the first year (September 2018 to August 2019) no matter how they were detected (opportunistic or active searches). We observed a similar tendency in the neighbouring Belgian zones sharing similar epidemiological context (ZOR_NO and ZOR_SUD), with 83% of the total number of carcasses being detected during the first year (data not shown).

Opportunistic surveillance (SAGIR) detected 85% of the carcasses in the Level III area (46/54), with most reports made by hunters (37%), farmers or common citizens (22%), and ONCFS officers (15%). If you exclude roadkill (50% of the total number of carcasses detected), the opportunistic surveillance share decreases to 65% during the first year, increasing the share found due to active search activities.

#### Comparison Between Zones

Table 2 shows the numbers of carcasses detected per square kilometre of forest area for each zone. We observe some differences between the three French zones, with a similar number of carcasses detected per km^2^ of forest in the French ZBC and ZBS zones but a lower number detected in ZBN. Compared to Belgian areas, the number of carcasses detected per kilometre square of forest in ZBC and ZBS is similar to the Belgian ZOR_SUD but lower than in ZOR_NO. The ZOR_ZI_SO zone had the highest number of carcasses per kilometre square of forest area, but this zone had a different status to the others as it was declared infected between January 2019 and May 2020.

**Table 2:** Surveillance efficacy indicators for different surveillance zones in bordering areas of France and Belgium (refer to Figure 1 for location of the zones).

**Country**	**Zone**	**Forest surface (ha)**	**No. of carcasses detected per 100 ha of forest area**	**No. of carcasses detected per 100 ha of forest area (excluding roadkill)**
Belgium	ZOR_NO	4,543	0.88	0.73
	ZOR_SUD	2,469	0.73	0.45
	**ZOR_total**	**7,012**	**0.83**	**0.63**
	**ZOR_ZI_SO**	**812**	**2.96**	**2.71**
France	ZBN	2,801	0.25	0.14
	ZBC	2,021	0.54	0.40
	ZBS	4,859	0.49	0.27
	**Total Level III area**	**9,681**	**0.46**	**0.26**

## Discussion

### Field Implementation

As presented in the results, we had to adapt the systematic combing protocol because it was arduous to implement and because it was very demanding in terms of human resources. We decided to target forest areas where dead WBs were most likely to be found using the model developed by (4). In a non-infected area at risk of introduction, the priority is to detect a case early. Thus, it is acceptable to target field search activities using a risk-based approach. Conversely, systematic combing is key to the ASF control strategy in an infected area as carcasses have to be detected and removed from the environment to stop transmission between animals (6).

The results also show that the number of hunter patrols was not steady over the months despite being planned on a weekly basis. Different hypotheses can be put forward. Firstly, although this activity was compensated, hunters were involved on a voluntary basis, so they had no obligation to do the patrols and had to cope with their professional and personal constraints. Secondly, we also perceived it was difficult to keep them motivated across the entire period. Proposed explanations based on feedback we received are that:

- providing appropriate feedback to field actors is a key issue and is never perfect in a crisis context.
- a changing agenda makes it difficult to prepare well. For instance, in the changing and uncertain epidemiological context, we were not able to draw up a long-term plan for surveillance activities from the beginning: hunter patrols initially planned for a few weeks had to be maintained for 2 years.
- policy decisions related to the hunting ban or financial compensation for hunting societies negatively affected communication with hunters and sometimes the data reports.
- delay in paying the patrols similarly complicated communication. Administrative procedures for such payments need to be better anticipated in the future.
- it was also difficult for volunteers to understand the need to report their field sessions on a weekly basis when hunting was re-opened.
- as the expected results in most cases were “no carcass found”, those implementing the searches might have experienced a feeling of failure. Our communication probably has to promote the objective of the fieldwork better.

### Reporting

Compiling data from different sources and using different spatial scales was not straightforward. It was thus decided to develop a shared database and an Android application supporting spatial data. A prototype was developed and tested using the KoBoToolbox platform (https://www.kobotoolbox.org/). Because it came too late in the programme, this tool was not routinely used in the end.

### Surveillance Efforts

By compiling surveillance efforts for all activities, we have a better picture of the true human resource involvement during this crisis. Nevertheless, this evaluation does not take into account the time spent on local or national coordination, nor the time spent on managing the carcasses (sampling and packaging in good biosecurity conditions). Those activities are, however, tricky points in the organisation of the surveillance activities. To complete this picture, a qualitative assessment of the perception of field actors would be necessary: as some activities were very demanding, we perceived that field teams were exhausted after a 1-year period.

### Surveillance Efficacy

Fifty-four carcasses were detected between September 2018 and the end of August 2020 in our Level III area, mainly due to opportunistic surveillance. Nevertheless, 50% of them were roadkill known to be less likely to be infected than other carcasses found in ASF-infected countries (10). Thus, they were recorded separately to give a more precise picture of the surveillance results. Without precise knowledge of the WB population, the question of our surveillance system's efficacy in detecting most of the carcasses is difficult to answer. Analysing data from the WB depopulation programme may help to do so. The hunting bag for the 2018–2019 season was 936 animals in the Level III area. In January 2019, fences were built, and a depopulation programme started. Thus, the WB population within the Level III area was under strict surveillance with limited opportunity to move outside the area. Over the 2019–2020 hunting season, the number of hunted WBs within the depopulation programme (by hunters and professionals) was 951 on April 19, 2020 (OFB data). On that date, the remaining population within this Level III area was estimated to be between 150 and 220 animals (based on field observations and data from camera traps—OFB data). Although it is probably imperfect, compiling this data gives a rough idea of the population level within this restricted zone of 300 km^2^. Guberti et al. (6) estimate that a desirable goal for dead WB surveillance is to report 10% of the carcasses. They estimated that natural mortality in WBs is 10% of the population. In our case, if we roughly estimate that 2,500 WBs lived in this area over the study period, around 250 animals may have died naturally. Our surveillance system succeeded in detecting 17% of this estimated dead WB population. In the context of a depopulation programme, the natural mortality percentage is probably even lower due to the intense hunting pressure, and as the depopulation progressed, it became more difficult to found one carcass.

Another way to evaluate our surveillance activities was to compare the French and Belgian surveillance data in similar areas (Belgian ZOR neighbouring the French border). We can hypothesise that the WB population was shared to some extent between the two countries (especially when a forest lays on both sides of the border). Thus, we used a proxy for this population (surface area of the forest) to calculate an indicator of the surveillance efficacy and we observed increased detection of carcasses in proximity to the ASF cases. Thus, proportionally to the forest surface area, more dead WBs were detected in France in ZBC and ZBS compared to ZBN, the farthest zone from the epizootic front. The difference is not only explained by more intense active search activities in ZBC and ZBS. Indeed, the opportunistic surveillance also detected fewer carcasses in ZBN proportionally to the forest surface area. We can hypothesise that the landscape may have influenced observation and thus reporting of carcasses in ZBN (more dense forest area with restricted public access). Nevertheless, field actors' lower awareness cannot be excluded.

Similarly, more dead WBs were detected in the Belgian zones closest to the epizootic front. Proportionally to the forest surface area, more dead WBs were detected in the Belgian ZOR_NO zone compared to ZOR_SUD, not directly in continuity with the infected forest. Similarly, ZOR_ZI_SO, which was classified as an infected area from January 2019 to May 2020, had the highest number of carcasses detected despite only a few (8) confirmed infected cases.

Finally, we note that active search activities interestingly supplement the results of opportunistic surveillance. For instance, between January and April 2019, 50% of the carcasses (excluding roadkill) were detected by active surveillance. Although, in the end, hunter patrols did not detect many carcasses compared to the time they spent in the field, they were a guarantee that no abnormal mass mortality occurred.

## Conclusion

France remained free from disease, and Belgium regained its free status in November 2020 (11). Despite the proximity between the Belgian infected area and the French border, no regulated zones as defined by the Commission Implementing Decision 2014/709/EU were decided for the French territory. Regular and detailed reporting of surveillance activities on the WB population, together with the depopulation programme in the Level III area, contributed to supporting this free status.

Surveillance of an epizootic in wildlife is always challenging. In this experience, we had to increase the field presence to actively detect new carcasses in a changing epidemiological situation. Those efforts contributed to increasing the number of carcasses detected. They were also a guarantee that no abnormal mass mortality occurred in the WB population.

The study of the surveillance effort and the comparison of the number of carcasses detected by surface area of forest is a first step in the evaluation of the surveillance activities undertaken during this crisis. In order to improve this evaluation, we are planning to organise a field experiment to compare, within an experimental plan, the efficacy of our different active search methods in controlled conditions. The criteria to be controlled relate to visibility and accessibility for the observers (the landscape is being modelled according to those criteria). We also plan to deepen our analysis on the carcass distribution and to better explore the probability of detection by comparing different surveillance efforts in the Level III area in France and the equivalent area in Belgium.

## Data Availability Statement

The datasets presented in this article are not readily available because their are a share property of OFB, FNC and Ministry of Agriculture. Requests to access the datasets should be directed to the corresponding author.

## Ethics Statement

Ethical review and approval was not required for the animal study because All animals tested were animal found dead.

## Author Contributions

SD, PC, EF, AD, and SR: active search protocol design. SD, CU, PC, and GP: data compiling. SD: data analysis. SD, GG, PC, EF, and TP: paper writing. SD, CU, TP, PC, GG, AD, ER, J-YC, GP, EF, and SR: critical revision of the manuscript. All authors contributed to the article and approved the submitted version.

## Conflict of Interest

The authors declare that the research was conducted in the absence of any commercial or financial relationships that could be construed as a potential conflict of interest.
